# Diabetes and gout: efficacy and safety of febuxostat and allopurinol

**DOI:** 10.1111/dom.12135

**Published:** 2013-06-12

**Authors:** M A Becker, P A MacDonald, B J Hunt, R L Jackson

**Affiliations:** 1 Pritzker School of Medicine, The University of ChicagoChicago, IL, USA; 2 Takeda Global Research & Development Center, Inc.Deerfield, IL, USA

**Keywords:** clinical trial, diabetes mellitus, drug utilisation

## Abstract

**Aim** Assess influences of demographics and co-morbidities of gout patients with or without diabetes on safety and efficacy of urate-lowering agents.

**Methods** Post-hoc analysis of 312 diabetic and 1957 non-diabetic gout patients [baseline serum urate levels (sUA) ≥8.0 mg/dl] enrolled in a 6-month randomized controlled trial comparing urate-lowering efficacy (ULE) and safety of daily xanthine oxidase inhibitors (XOIs) febuxostat (40 mg or 80 mg) and allopurinol (200 mg or 300 mg). We compared baseline demographic, gout and co-morbid characteristics, ULE, and safety of XOI treatment in diabetic and non-diabetic gout patients. ULE was measured by the proportion of diabetic and non-diabetic patients in each treatment group achieving final visit sUA < 6.0 mg/dl. Safety was monitored throughout the trial.

**Results** Diabetic gout patients were older, more frequently female, and had longer gout duration. Co-morbidities were more frequent among diabetic patients: cardiovascular disease; impaired renal function; hyperlipidemia; and obesity (body mass index >30 kg/m^2^) (p < 0.001 for all comparisons). Febuxostat 80 mg ULE exceeded that of febuxostat 40 mg or allopurinol (p < 0.050) at all levels of renal function, achieving sUA goal range in the majority of diabetic and non-diabetic patients. Diabetics and non-diabetics reported self-limiting diarrhoea and URIs as the most common adverse events.

**Conclusions** Despite higher co-morbidity rates in diabetic patients, febuxostat and allopurinol were safe in both groups at the doses tested. Febuxostat 80 mg achieved sUA <6.0 mg/dl more often than febuxostat 40 mg or allopurinol at commonly prescribed doses.

## Introduction

A key aim in management of gout (monosodium urate crystal deposition disease) is achievement and long-term maintenance of serum urate levels (sUA) in a sub-saturating range, most commonly recommended as <6.0 mg/dl 1–2. Substantial evidence confirms the view that accomplishment of this aim is associated with the prevention and reversal of urate crystal deposition 3–4, and ultimately, with cessation/reversal of gout signs 5–6 and symptoms 6–9.

Among potential impediments to successful gout management are the significant metabolic, cardiovascular (CV) and renal co-morbidities that are common among gout patients 10–14 and may influence the safety and/or efficacy of available gout therapies. The co-existence of chronic kidney disease (CKD) and gout 14 provides examples of such influences. Moderate or more advanced kidney disease increases the risk for further renal impairment when non-steroidal anti-inflammatory drugs are administered to treat gout flares or for flare prophylaxis, as well as severely reducing the urate-lowering efficacy (ULE) of the uricosuric agent probenecid. Similarly, reduction in the daily dose of allopurinol, the most commonly prescribed urate-lowering agent, has long been advocated 15 and widely adopted 16, though never formally validated 17,18 and only recently rejected 2, as a means of avoiding severe allopurinol toxic reactions in gout patients with impaired creatinine clearances.

An association of gout with diabetes mellitus was noted more than a century ago and has been reaffirmed frequently 20–21. Mechanisms involving genetic, environmental and physiological interactions 22–23 have been proposed to account for this association, but a unitary explanation has yet to be identified. Nevertheless, management of gout in diabetic patients presents a challenge because of the substantially greater prevalence of each co-morbidity in patients with gout or with diabetes compared with non-gouty and non-diabetic individuals 11,12. We have, therefore, asked whether concomitant gout and diabetes influences the efficacy or safety of xanthine oxidase inhibitors (XOIs). A large dataset gathered in a previously reported randomized, double-blind trial comparing urate-lowering treatment with febuxostat or allopurinol 25 afforded the opportunity for post-hoc comparisons of diabetic and non-diabetic gout patients with regard to: baseline demographic, gout-related and co-morbid characteristics; and urate-lowering effectiveness and tolerability of XOIs.

## Materials and Methods

### Patients

Patients age 18–85 years with a diagnosis of gout fulfilling American Rheumatology Association preliminary criteria 26 and with baseline sUA ≥8.0 mg/dl were eligible for enrollment in the 6-month CONFIRMS trial comparing the safety and ULE of febuxostat and allopurinol 25. Exclusion criteria included severe GFR impairment [defined as baseline estimated creatinine clearance (eCL_cr_) <30 ml/min 27, calculated by the Cockcroft–Gault formula corrected for ideal body weight 28–29). Diabetic patients with gout enrolled in the CONFIRMS trial were identified post-hoc by a history of a physician diagnosis of diabetes.

### Study Procedures

Patients were enrolled at 324 United States' sites. Institutional Review Board approval was obtained for each site, and all patients provided written informed consent and Health Insurance Portability and Accountability Act authorization prior to study-related procedures.

Patients receiving urate-lowering pharmacotherapy at screening discontinued such treatment at least 30 days before randomization. Patient screening evaluations included: physical examination and vital signs; medical history; completion of a pre-specified CV history/risk form; laboratory tests (sUA, comprehensive chemistry panel, haematology, urinalysis, and, for women, pregnancy test); electrocardiogram (EKG); assessment for tophi and gout flare; and concomitant medication use. Safety was evaluated at all visits. sUA values were blinded after the baseline (qualifying) determination at day−4.

Patients were randomized 1 : 1 : 1 on day 1 to receive daily febuxostat 40 mg, febuxostat 80 mg, or allopurinol (Apotex, Weston, FL, USA). Among patients randomized to allopurinol, those with normal renal function (eCLcr ≥ 90 ml/min) or mild renal impairment (eCLcr 60–89 ml/min) received 300 mg daily and those with moderate renal impairment (eCLcr 30–59 ml/min) received 200 mg daily 15. Randomization was stratified by baseline renal function and prior completion of either of two long-term open-label XOI treatment trials 7–9. The doses of allopurinol were chosen to reflect those commonly prescribed in clinical practice, 95% of which are ≤300 mg daily 30.

All patients received prophylaxis for gout flares, with either colchicine 0.6 mg daily, or naproxen 250 mg twice daily (both Westward Pharmaceutical Corporation, Eatontown, NJ, USA) throughout the 6-month treatment period. Patients with eCL_cr_ <50 ml/min were permitted only colchicine prophylaxis. All patients receiving naproxen also received lansoprazole 15 mg daily (Takeda Global Research & Development Center, Inc, Deerfield, IL, USA).

The proportion of diabetic and non-diabetic patients in each treatment group achieving target sUA <6.0 mg/dl at final visit was the efficacy outcome of primary interest. The proportion of patients in each treatment group with mild and with moderate renal impairment and a final visit sUA <6.0 mg/dl were additional efficacy endpoints. Safety in diabetic and non-diabetic patients was also compared across treatment groups. Acute gout flares requiring treatment were not considered adverse events (AEs). AEs were collected at each visit throughout the study and were coded using Medical Dictionary for Regulatory Activities (MedRA) terminology. All deaths and AEs considered potentially CV-related were reviewed by a Cardiovascular Endpoints Committee composed of three blinded expert adjudicators who determined if the AE met Antiplatelet Trialists Collaboration criteria for APTC or non-APTC CV events 31–32.

Statistical analyses of the efficacy endpoints and AE rates for the overall study have been previously described 25. To determine statistically significant differences between diabetic and non-diabetic subjects for baseline characteristics of age, body mass index (BMI), baseline sUA and years with gout, analysis of variance was used; for all other categorical baseline variables, Fisher's exact test was used. In addition, Fisher's exact test was used to determine statistically significant differences between diabetic and non-diabetic subjects within each treatment group in the proportions of subjects who achieved the primary and additional efficacy endpoints and in rates of incident AEs.

Primary endpoint and additional efficacy analyses were performed on a modified intent-to-treat (mITT) population (N = 2268), defined as all randomized patients with baseline sUA ≥8.0 mg/dl who received at least one dose of study drug. One non-diabetic patient randomized to allopurinol was withdrawn from the study after one dose because the qualifying baseline sUA was <8.0 mg/dl. Safety analyses were carried out on all randomized patients receiving at least one dose of study drug (ITT group; N = 2269).

## Results

Of 312 diabetic patients identified among 2269 gout patients, 89, 113 and 110 patients were randomized to receive daily febuxostat 40 mg, febuxostat 80 mg and allopurinol 300 or 200 mg, respectively. Among diabetic gout patients randomized to receive allopurinol according to baseline renal function, 69 received 300 mg and 41 received 200 mg daily.

There were no statistically significant differences across treatment groups for either diabetic or non-diabetic patients with respect to the distribution of baseline patient demographic characteristics (data not shown). Compared with 1957 non-diabetic gout patients, diabetic gout patients were less likely to be male (87.5% vs. 95.5%; p < 0.001), or white (73.4% vs. 83.5%; p < 0.001) or to use alcohol (52.2% vs. 70.8%; p < 0.001). Diabetic gout patients were more likely than non-diabetic gout patients to be older (mean age 58.2 vs. 52.0 years; p < 0.001) and have a BMI ≥30 kg/m^2^ (78.5% vs. 61.2%; p < 0.001) (Table1).

**Table 1:** Demographics, baseline characteristics and co-morbidities of diabetic and non-diabetic patients in CONFIRMS*

Variable	Diabetic subjects, N = 312	Non-diabetic subjects, N = 1957
Male, n (%)	273 (87.5)	1868 (95.5)
Race, n (%)		
American Indian or Alaska Native	4 (1.3)	18 (0.9)
Asian	14 (4.5)	74 (3.8)
Black or African American	57 (18.3)	171 (8.7)
Native Hawaiian or other Pacific Islander	6 (1.9)	26 (1.3)
White	229 (73.4)	1634 (83.5)
Other	2 (0.6)	32 (1.6)
Missing	0	2 (0.1)
Ethnicity, n (%)		
Hispanic or Latino	18 (5.8)	131 (6.7)
Not Hispanic or Latino	294 (94.2)	1825 (93.3)
Missing	0	1 (0.1)
Age, years		
Mean ± s.d.	58.2 ± 11.3	52.0 ± 11.6
Range	19–85	19–85
Body mass index (kg/m^2^)		
≥30, n (%)	245 (78.5)	1197 (61.2)
Mean ± s.d.	36.0 ± 7.51	32.3 ± 5.97
Range	21–64	16–64
Alcohol use† , n (%)	163 (52.2)	1386 (70.8)
Serum urate (mg/dl), n (%)		
<9.0	116 (37.2)	721 (36.8)
9.0– < 10.0	88 (28.2)	611 (31.2)
10.0– < 11.0	60 (19.2)	394 (20.1)
11.0– < 12.0	34 (10.9)	155 (7.9)
≥12.0	14 (4.5)	76 (3.9)
Mean ± s.d.	9.6 ± 1.23	9.6 ± 1.17
Range	8–14	8–15
Years with gouty arthritis		
Mean ± s.d.	12.8 ± 10.1	11.4 ± 9.2
Range	0–44	0–53
Presence of tophi, n (%)	57 (18.3)	421 (21.5)
Renal function‡, n (%)		
Moderate impairment	112 (35.9)	290 (14.8)
Mild impairment	133 (42.6)	948 (48.4)
Normal	67 (21.5)	719 (36.7)
Medical history, n (%)		
Any cardiovascular disease	269 (86.2)	1028 (52.5)
Hypertension	258 (82.7)	941 (48.1)
Hypercholesterolemia	30 (9.6)	132 (6.7)
Hyperlipidemia	203 (65.1)	739 (37.8)
Use of low-dose aspirin (≤325 mg daily)	111 (35.6)	294 (15.0)

* There are no statistically significant differences among treatment groups with respect to the distribution of baseline characteristics.

† Alcohol use was defined as 1–14 drinks per week.

‡ Moderate baseline renal impairment: estimated creatinine clearance (eCLcr) 30 to <60 ml/min; mild baseline renal impairment: eCLcr 60 to <90 ml/min; normal: eCLcr ≥90 ml/min.

Baseline co-morbidities were common in both diabetic and non-diabetic gout patients (Table1), but several were present at significantly higher rates among diabetics. This was the case for: CV disease (86.2% vs. 52.5%; p < 0.001), including: hypertension [82.7% vs. 48.1%; p < 0.001], coronary artery disease [22.1% vs. 6.3; p < 0.001], cardiac arrhythmias [18.3% vs. 8.9%; p < 0.001] and myocardial infarction [9.9% vs. 3.0%; p < 0.001]); impaired renal function (eCLcr <90 ml/min; 78.5% vs. 63.3; p < 0.001); and hyperlipidemia (65.1% vs. 37.8%; p < 0.001).

Diabetic and non-diabetic gout patients did not differ in either baseline mean sUA or the proportion of patients with baseline tophi (Table1). The mean duration of gout was, however, longer in the diabetic (12.8 years) compared with the non-diabetic (11.4 years; p = 0.021) cohort. At baseline, the majority of diabetic gout patients were receiving either insulin or oral hypoglycaemic agents (225/312; 72.1%).

When the demographics of diabetic and non-diabetic patient groups were compared by renal function status, two differences were noted. First, rates of low-dose aspirin use were higher in patients with moderate renal impairment than in patients with normal renal function (50.0% vs. 19.4%, respectively, in diabetic gout patients; 29.0% vs. 8.2%, respectively, in non-diabetic gout patients). Second, higher proportions of both diabetic and non-diabetic patients with moderate renal impairment (compared with patients with normal renal function) had tophi: 20.5% vs. 13.4% for diabetic gout patients; 27.2% vs. 18.9% for non-diabetic gout patients).

**Figure 1:**
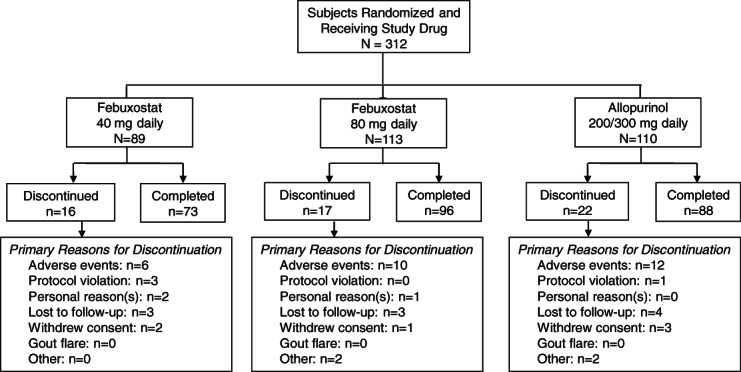
Flow of diabetic gout patients randomized and receiving study drug in the CONFIRMS trial.

Premature study discontinuation (figure 1) occurred in 55 (17.6%) diabetic patients across the febuxostat 40 mg, febuxostat 80 mg and allopurinol treatment groups (16, 17 and 22 patients, respectively), compared with 363 (18.5%) non-diabetic patients (109, 141 and 113 patients, respectively). The most common primary reason for discontinuation in all treatment groups among both diabetic and non-diabetic gout patients was an AE (9.0 % and 7.5%, respectively).

### Efficacy

**Figure 2:**
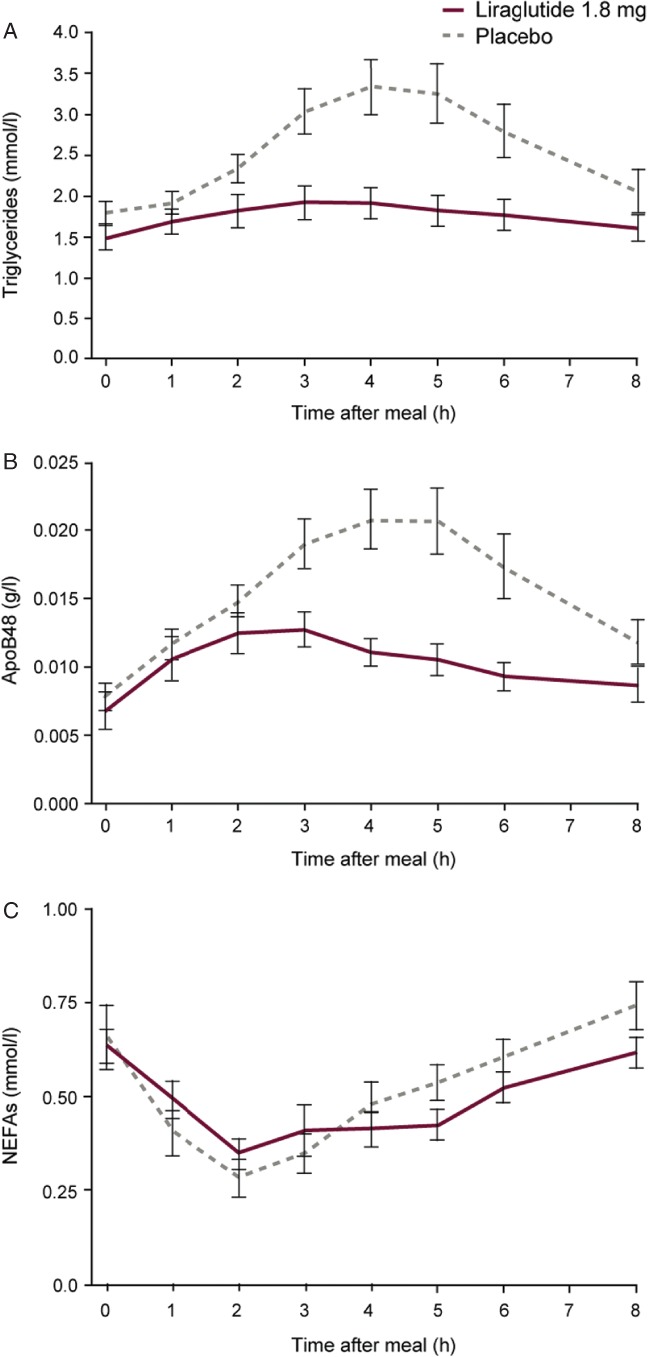
Achievement of sUA <6.0 mg/dl. FEB, febuxostat; ALLO, allopurinol. (a) all patients: ^a^p < 0.001 for comparisons between FEB 80 mg and either FEB 40 mg or ALLO in diabetic patients; ^b^p < 0.001 for comparisons between FEB 80 mg and either FEB 40 mg or ALLO in non-diabetic patients; ^c^p < 0.050 for comparison between diabetic and non-diabetic subjects receiving ALLO. (b) Patients with mild renal impairment: ^a^p < 0.050 for comparisons between FEB 80 mg and either FEB 40 mg or ALLO in diabetic patients; ^b^p < 0.001 for comparisons between FEB 80 mg and either FEB 40 mg or ALLO in non-diabetic patients; ^c^2 patients with mild renal impairment received 200 mg ALLO. (c) Patients with moderate renal impairment: ^a^p < 0.001 for comparisons between FEB 80 mg and either FEB 40 mg or ALLO in diabetic patients; ^b^p < 0.050 for comparisons between FEB 80 mg and either FEB 40 mg or ALLO in non-diabetic patients; ^c^p < 0.050 for comparison between diabetic and non-diabetic patients receiving FEB 40 mg; ^d^p < 0.050 for comparison between diabetic and non-diabetic subjects receiving FEB 80 mg; ^e^1 patient with moderate renal impairment received 300 mg ALLO.

The ULE of febuxostat 80 mg in both diabetics and non-diabetics was superior to that of either febuxostat 40 mg or allopurinol (figure 2), and this finding held for comparisons involving all patients (figure 2A) as well as patients with either mild (figure 2B) or moderate (figure 2C) renal impairment (p < 0.050 for all comparisons of febuxostat 80 mg with either febuxostat 40 mg or allopurinol). In both the diabetic and non-diabetic gout patients, the proportions of all patients (figure 2A) or patients with mild (figure 2B) or with moderate (figure 2C) impairment of renal function who achieved final visit sUA <6.0 mg/dl with febuxostat 40 mg and allopurinol were comparable.

Among patients with moderate renal impairment (figure 2C), however, febuxostat 40 mg showed lower efficacy in diabetic than in non-diabetic gout patients (p < 0.05), while febuxostat 80 mg showed higher efficacy (p < 0.05).

With allopurinol treatment, lower ULE was observed in the entire group of diabetic vs. non-diabetic gout patients (p = 0.021) (figure 2A), but this finding is likely attributable to the higher proportion of patients with moderate renal impairment in the diabetic than in the non-diabetic gout patient groups (Table1), which, by protocol, mandated allopurinol dose assignment of 200 mg daily (vs. 300 mg daily) when estimated eGFR was <60 ml/min. The numerical difference in efficacy between diabetic and non-diabetic patients with moderate renal impairment assigned allopurinol (figure 2C) was not statistically significant (p = 0.161).

### Safety

At least one AE was reported in 46, 62 and 66% of diabetic gout patients receiving febuxostat 40 mg, febuxostat 80 mg, and allopurinol 300 or 200 mg, respectively (Table2). Incidences of AEs among non-diabetic patients were 58, 53 and 56% in the respective treatment groups. Self-limited diarrhoea, upper respiratory tract infections, musculoskeletal or joint signs and symptoms, and abnormal liver function analyses were among the most common AEs across treatment groups in both diabetic and non-diabetic gout patients.

**Table 2:** Adverse events

	All diabetic patients, N = 312	All non-diabetic patients, N = 1957
	n (%)		
Total patients with ≥1 AE	184 (59)	1088 (56)
Most frequently* reported AEs		
Diarrhoea	29 (9)	120 (6)
Upper respiratory tract infections	26 (8)	155 (8)
Musculoskeletal and connective tissue signs and symptoms	22 (7)	91 (5)
Joint related signs and symptoms	13 (4)	74 (4)
Liver function analyses	15 (5)	150 (8)
Dermatitis and eczema	9 (3)	44 (2)
Lower respiratory tract infections	9 (3)	31 (2)
Nausea and vomiting	13 (4)	39 (2)
All serious AEs†		
Blood and lymphatic system disorders	0	1 (<1)
Cardiac disorders	3 (<1)	11 (1)
Ear and labyrinth disorders	0	1 (<0.1)
Gastrointestinal disorders	3 (<1)	6 (<1)
General disorders and administration site conditions	2 (<1)	3 (<1)
Hepatobiliary disorders	2 (<1)	1 (<1)
Immune system disorders	0	2 (<1)
Infections and infestations	5 (2)	8 (<1)
Injury, poisoning, and procedural complications	1 (<1)	6 (<1)
Metabolism and nutrition disorders	1 (<1)	2 (<1)
Musculoskeletal and connective tissue disorders	1 (<1)	2 (<1)
Neoplasms—benign, malignant and unspecified	1 (<1)	9 (1)
Nervous system disorders	1 (<1)	9 (1)
Renal and urinary disorders	0	2 (<1)
Reproductive system and breast disorders	1 (<1)	0
Respiratory, thoracic, and mediastinal disorders	2 (<1)	1 (<1)
Vascular disorders	0	2 (<1)
Elevated serum LFTs	n/N (%)		
ALT		
≥2 X ULN	21/282 (7)	172/1819 (9)
≥3 X ULN	3/282 (1)	51/1819 (3)
AST		
≥2 X ULN	16/282 (6)	93/1818 (5)
≥3 X ULN	2/282(<1)	26/1818 (1)
ALT and AST concurrently		
≥3 X ULN	2/282 (<1)	17/1818 (1)

AE, adverse event; ALT, alanine aminotransferase; AST, aspartate aminotransferase; LFT, liver function tests; ULN, upper limit of normal.

* Events reported in high level terms in ≥5% of patients in any treatment group.

† Serious AEs listed by system organ class.

Serious AEs occurred in 1 (1%), 8 (7%) and 8 (7%) of diabetic gout patients in the febuxostat 40 mg, febuxostat 80 mg, and allopurinol 300/200 mg treatment groups, respectively (Table 2), compared with 18 (3%), 20 (3%) and 23 (4%) of non-diabetic gout patients randomized to the respective treatment groups. No category of serious AE occurred in more than one diabetic gout patient in any treatment group, with the exception of infections and infestations (3 or 3% of allopurinol-treated and 2 or 2% of febuxostat 80 mg-treated patients) and cardiac disorders, gastrointestinal disorders and hepatobiliary disorders (each occurring in 2 or 2% of allopurinol-treated patients).

Six APTC events (0.3%) occurred among 2269 enrolled patients: three receiving febuxostat 80 mg, three receiving allopurinol. Of patients with an APTC event, one was diabetic. This patient, who received febuxostat 80 mg, had a serious adverse reaction of non-fatal myocardial infarction. Five deaths occurred among patients enrolled in the study, including a 48 year old diabetic man on febuxostat 80 mg. Death was attributed to brain edema and obstructive pulmonary disease.

Elevations in alanine aminotransferase (ALT) and aspartate aminotransferase (AST) levels are listed in Table2. No patient had concurrent elevations in ALT or AST and bilirubin or alkaline phosphatase. Non-fasting blood glucose levels remained stable in diabetic patients; mean changes (±s.d.) from baseline to final visit in the febuxostat 40 mg, febuxostat 80 mg, and allopurinol groups were 7 (±59) mg/dl, 6 (±55) mg/dl and 6 (±41) mg/dl, respectively.

## Discussion

This post-hoc analysis is the first detailing the baseline characteristics of diabetic gout patients compared with non-diabetic gout patients and examining the ULE and safety of XOI therapy in these two groups. We confirmed high rates of baseline metabolic, CV and renal disorders in both patient groups, which were, however, significantly greater among diabetic gout patients. To what extent differences in co-morbidity rates reflect differences in demographic characteristics of these two groups, such as age, gender, BMI or duration of gout, is unclear. However, an alternative view, that gout and diabetes each contribute at least in part independently to the co-morbid burden, remains a possibility to be delineated in prospective trials in which patients are randomized specifically to compare more equivalent numbers of diabetic and non-diabetic gout patients. Nevertheless, despite higher co-morbidity rates in diabetic than non-diabetic gout patients, treatment with febuxostat (40 mg or 80 mg) or allopurinol (300/200 mg) had similar safety profiles.

In the case of patients with moderate renal impairment, the ULE of febuxostat 40 mg and allopurinol 200 mg was lower in diabetic compared with non-diabetic gout patients. Although the numbers of diabetic gout patients treated with low doses of XOIs are small (and, in the case of allopurinol, dose reduction was prescribed for patients with this level of CKD), reduction in the urate-lowering response to low doses of XOIs in diabetic gout patients with more advanced renal impairment is suggested. Whether this reduction reflects particular functional deficits in the kidneys of diabetic patients with gout or is a consequence of other factors in the management of such patients is uncertain. For example, low doses of aspirin, prescribed in half of the diabetic patients with moderate renal impairment studied here, have a uricoretentive effect, which may explain at least in part the failure of low-dose febuxostat or allopurinol treatment to achieve goal range urate levels. Of interest, among diabetic patients with moderate renal impairment receiving the higher dose (80 mg) of febuxostat, achievement of goal range serum urate was substantial and at least equivalent to that observed in non-diabetic patients treated with this dose of febuxostat. Whether higher doses of allopurinol than studied here would similarly improve achievement of the urate-lowering goal range in such patients is an important but unanswered question.

Two limitations to the interpretation of our study results warrant mention. First, our results were obtained with clinical practice allopurinol dosing patterns 30–33 rather than with newly proposed dosing recommendations 2. The doses of allopurinol selected for the CONFIRMS trial 25 were fixed and renal function-adjusted 15 to reflect those most commonly prescribed in clinical practice in the United States and E.U. at trial inception 30 and as recently as 2012 33. The 2012 American College of Rheumatology guidelines for urate-lowering in gout patients 2, however, recommend titration of allopurinol daily dose to achievement of sUA <6.0 mg/dl, despite expressing concern with the available long-term safety data for allopurinol dosing >300 mg daily, particularly in patients with significant renal impairment. Second, we did not evaluate flare reduction and tophus resolution of febuxostat and allopurinol urate-lowering treatment because prior studies with these agents 5–9 have indicated that clinical benefits require longer term maintenance of sub-saturating urate levels 7 than the 6 months of the current trial.

Febuxostat 80 mg achieved goal range sUA <6.0 mg/dl more often than febuxostat 40 mg or allopurinol at doses commonly used in clinical practice, supporting recommendations for monitoring the sUA response to the initial doses of either XOI and titrating daily doses until goal range urate has been achieved 1–2. As a matter of practical significance, the reduced response rates of diabetic patients with moderate renal impairment to febuxostat 40 mg or allopurinol 200 mg should alert the clinician to the likely need for dose titration to reach goal urate levels in such patients.
